# Mycetoma: Experience of 482 Cases in a Single Center in Mexico

**DOI:** 10.1371/journal.pntd.0003102

**Published:** 2014-08-21

**Authors:** Alexandro Bonifaz, Andrés Tirado-Sánchez, Luz Calderón, Amado Saúl, Javier Araiza, Marco Hernández, Gloria M. González, Rosa María Ponce

**Affiliations:** 1 Department of Mycology, Dermatology Service, General Hospital of Mexico, Mexico City, Mexico; 2 Departamento de Microbiología, Facultad de Medicina, Universidad Autónoma de Nuevo León, Monterrey, Nuevo León, Mexico; University of Tennessee, United States of America

## Abstract

Mycetoma is a chronic granulomatous disease. It is classified into eumycetoma caused by fungi and actinomycetoma due to filamentous actinomycetes. Mycetoma can be found in geographic areas in close proximity to the Tropic of Cancer. Mexico is one of the countries in which this disease is highly endemic. In this retrospective study we report epidemiologic, clinical and microbiologic data of mycetoma observed in the General Hospital of Mexico in a 33 year-period (1980 to 2013). A total of 482 cases were included which were clinical and microbiology confirmed. Four hundred and forty four cases (92.11%) were actinomycetomas and 38 cases (7.88%) were eumycetomas. Most patients were agricultural workers; there was a male predominance with a sex ratio of 3∶1. The mean age was 34.5 years old (most ranged from 21 to 40 years). The main affected localization was lower and upper limbs (70.74% and 14.52% respectively). Most of the patients came from humid tropical areas (Morelos, Guerrero and Hidalgo were the regions commonly reported). The main clinical presentation was as tumor-like soft tissue swelling with draining sinuses (97.1%). Grains were observed in all the cases. The principal causative agents for actinomycetoma were: *Nocardia brasiliensis* (78.21%) and *Actinomadura madurae* (8.7%); meanwhile, for eumycetomas: *Madurella mycetomatis* and *Scedosporium boydii* (synonym: *Pseudallescheria boydii*) were identified. This is a single-center, with long-follow up, cross-sectional study that allows determining the prevalence and characteristics of mycetoma in different regions of Mexico.

## Author Summary

Mycetoma is a chronic, subcutaneous granulomatous disease that usually begins after traumatic inoculation with causative microorganisms. Based on its etiology, mycetoma is referred to eumycetoma when the infection is caused by filamentous fungi, and actinomycetoma when the infection is due to aerobic actinomycetes (in Mexico predominantly *Nocardia brasiliensis*). Establishing the etiology is extremely important since it impacts treatment regimens. Mycetoma typically presents around the Tropic of Cancer between latitude 15° South and 30° North (also known as “mycetoma belt”) affecting poor populations in Africa, Asia, and Latin America, including Mexico, which represents a highly endemic area with higher frequencies of actinomycetomas. Mycetoma usually affects males (male∶female ratio of 3∶1), agricultural or rural workers (age range 20–40 years) that typically do not have access to protective equipment. The main clinical presentation is as soft tissue swelling with sinus tract formation draining grains, which leads to diagnosis. The foot is the most commonly affected localization; however, when disease presents in high risk areas, such as the trunk, it can disseminate to the lungs and spinal cord. This report represents a single center study which provides epidemiologic, clinical, and microbiological data of mycetoma cases in different regions of Mexico.

## Introduction

Mycetoma is a chronic granulomatous disease, associated with a progressive, inflammatory reaction that clinically presents as tumor-like soft tissue swelling with sinus tract formation that drains purulent material containing grains. Mycetoma usually results of traumatic implantation of soil organisms on subcutaneous tissue; can be classified as eumycetoma or actinomycetoma depending on whether the infection is caused by filamentous fungi or aerobic filamentous actinomycetes, respectively [1], [2], [3].

Mycetoma represents a classical neglected disease that primarily affects the poorer populations and rural regions of Africa, Latin America, and Asia at latitudes defined as the “mycetoma belt” where higher mycetoma frequencies are observed. This region is located around the Tropic of Cancer, between latitudes 15° South and 30° North, encompassing the countries with the highest rates of infection including Sudan, Somalia, Senegal, India, Yemen, Mexico, and Venezuela [1], [4], [5]. The predominant climate of the “mycetoma belt” is subtropical and dry tropical with an annual average rainfall of about 500–1000 mm and temperatures ranged from 10–20°C to 20–40°C, respectively. This region is characterized by low humidity and low annual rainfall with well-defined alternating rainy and dry seasons. Actinomycetomas caused by *Nocardia* spp. occur mostly in regions with higher humidity, while actinomycetoma caused by *Actinomadura* spp. and *Streptomyces* spp. or eumycetoma occur in drier areas with low relative humidity [1], [3], [4], [5].

Most causative agents of mycetoma, including fungi and actinomycetes, have been isolated from soil, decaying organic matter, plants and thorns; and, the disease is usually associated with traumatic injury followed by inoculation of the microorganism propagule. There are three main factors associated with the establishment of disease: inoculum size, immune status of the host, and hormonal adaptation (based on the observation that men typically develop the disease) [1], [3], [6], [7], [8], [9].

Epidemiological data from different areas demonstrate that males are more affected (sex ratio 3–4∶1), ranging in age between the third and fourth decades of life (20–40 years). Some studies have reported that 3–5% of cases affect children committed to field-work [6], [10], [11]. Mycetoma is common in persons that work in rudimentary conditions without protective garments or shoes leading to the presentation of the illness primarily in poor rural workers or homemakers that participate in outdoor activities. Nearly all cases affect the lower limbs (75%), especially the foot and lower limbs. The nature of the patient's occupation also influences disease presentation, for example lumberjacks and sugarcane carriers generally present with mycetoma on the back [6], [10], [11].

The incubation period is unknown, disease symptoms present months to years after traumatic inoculation, depending on the inoculum size, strain virulence, and the host's immune response [7]. Because reporting mycetoma cases is not mandatory, the worldwide incidence is unknown; however, a recent published meta-analysis by van de Sande [11] reported incidence rates of 3.49 and 1.81 per 100,000 habitants in Mauritania and Sudan, respectively; while Mexico showed the highest rate in Latin American. Although a separate study reported that the number of cases per year in Sudan, Mauritania, and Mexico were 106, 80.7, and 73, respectively [6], several cases are probably not reported.

The objective in this study was to provide epidemiological, clinical and microbiological data of mycetoma in different regions on Mexico, presenting at a public single center, the General Hospital of Mexico (specialty hospital).

## Materials and Methods

The Institutional Review Board approved the retrospective (cross-sectional) analysis of the database and clinical records of the Mycology Department of the Dermatology Service at the General Hospital of Mexico, patients were enrolled between January 1980 and December 2013 (34 years). We included all cases of mycetoma confirmed by microscopic observation of grains by direct examination with 10% potassium hydroxide (KOH), saline solution, and lugol solution. The culture media used were Sabouraud dextrose agar and Yeast extract agar, however, when infection by *Actinomadura madurae* was suspected, Lowenstein-Jensen agar, and BHI agar (Brain Heart Infusion) were used. Histological examination was performed in some cases using hematoxylin and eosin (H&E), Grocott's methenamine silver (GMS), and Periodic acid–Schiff (PAS)

Actinomycetes identification was carried out using micro morphological criteria (Gram and Kinyoun stains) as well as biochemical and major phenotypic tests such as urease production, hydrolysis of casein, gelatin, tyrosine, xanthine, hypoxanthine substrates and, growth at 45°C [12], [13]. Fungal agent identification was based on morphological and reproductive form criteria and on biochemical tests. Some strains were identified using molecular techniques (by amplification and sequence analysis of ribosomal DNA. the internal transcribed spacer region (ITS), the translation elongation factor 1 alpha (*EF1-α*), the partial beta tubulin gene (*TUB*), and the small subunit of the nuclear ribosomal RNA gene [nucSSU]). General epidemiologic and clinical data were extracted from clinical records. All patients remained anonymous and descriptive statistics were used to analyze the data.

## Results

A total of 482 mycetoma cases were included in the present study, one patient presented simultaneously two different mycetomas. Demographic data are described in Table 1.

**Table 1:** Demographic data of the study population.

Variables	Number of cases (%)
**Type of Mycetoma**	
Actinomycetoma	444 (92.11)
Eumycetoma	38 (7.88)
Total	482 cases (100)
**Gender**	
Male	358 (74.27)
Female	124 (25.72)
Male∶female ratio	2.8∶1
**Evolution**	
Shortest	2 months
Longest	36 years
Average	2.2 years
**Anatomical localization**	
Lower limbs (feet, legs)	341 (70.74)
Upper limbs (hands, arms)	70 (14.52)
Trunk (anterior and posterior thorax, abdomen)	49 (10.16)
Head and neck	4 (0.82)
Several locations	10 (2.07)
Lymphatic dissemination (*e.g.*, foot-inguinal region)	8 (1.65)

**Figure 1:**
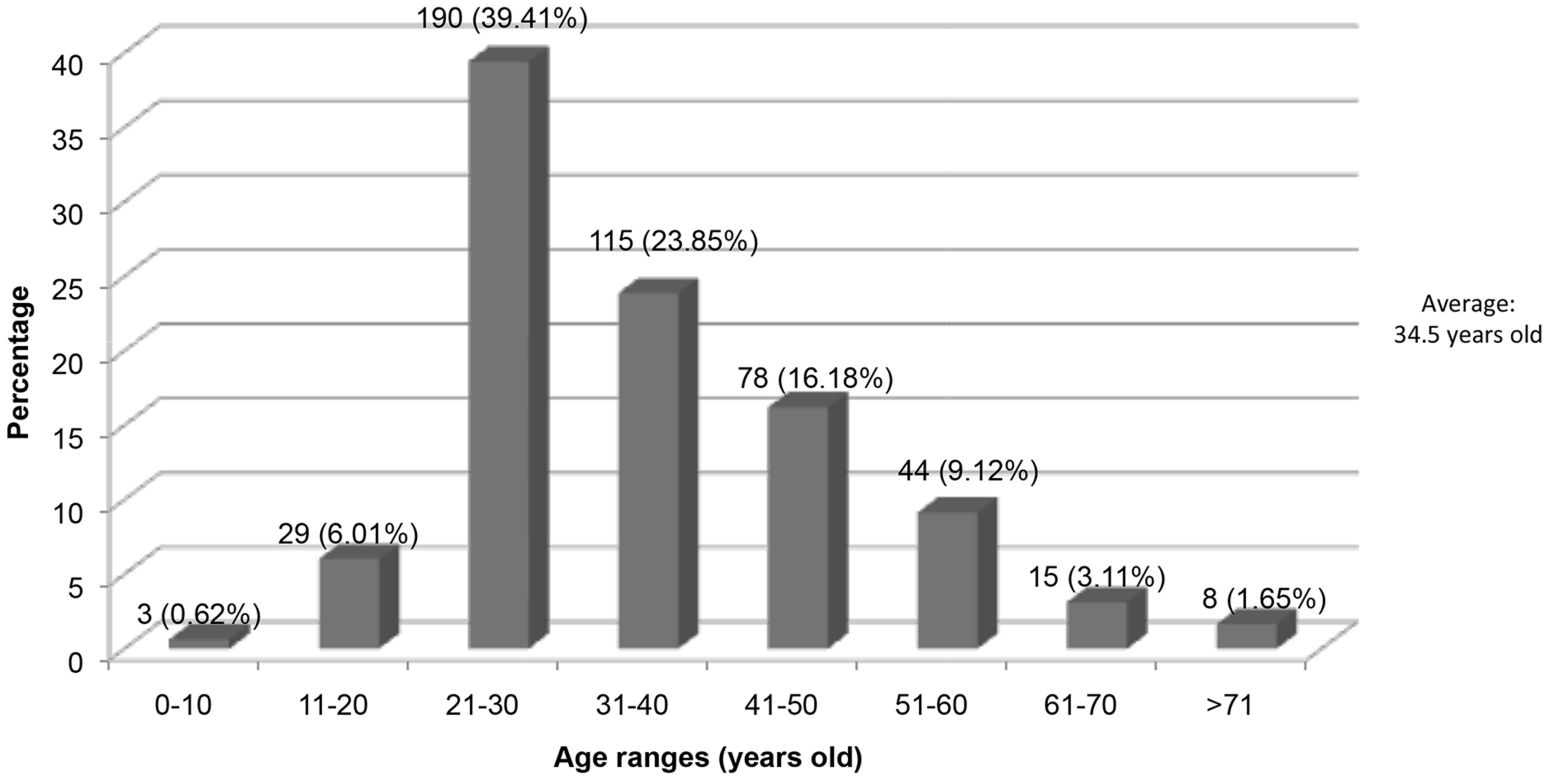
Age distribution of the mycetoma patients.

**Figure 2:**
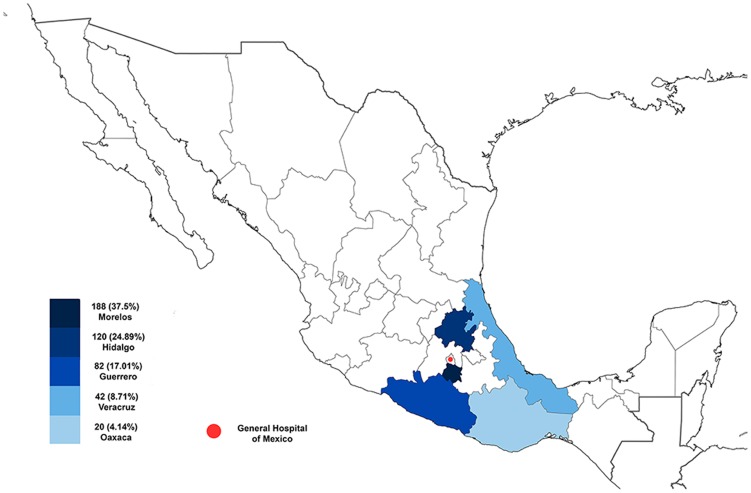
Geographical location of the five states with the higher incidence of mycetoma in Mexico.

Figure 1 illustrates the age distribution of mycetoma, and Figure 2 the geographic mycetoma distribution in Mexico. The Pacific Ocean zone including Morelos, Guerrero, and Oaxaca accounted for 284 cases (58.92%) and the Gulf of Mexico zone (including Hidalgo and Veracruz) accounted for 162 cases (33.60%). The remaining 36 cases presented in other states including Chiapas, Tabasco, Puebla, Michoacan, Colima, Jalisco, San Luis Potosi, Durango, Chihuahua, and Baja California.

Most cases were actinomycetomas (92.11%) with a male to female ratio of 2.8∶1. However, this ratio changes in cases due to *Actinomadura madurae*, of the 42 cases 13 (30.06%) were males and 29 (69.04%) were females with a male∶female ratio of 1∶2.2. Age ranges were classified in decades, the mean age was 34.5 years old (range 7–92 years). Pediatric cases (<18 years old) were 20/482 (4.14%) and 5/482 cases (1.083%) were younger than 15 years old.

**Figure 3:**
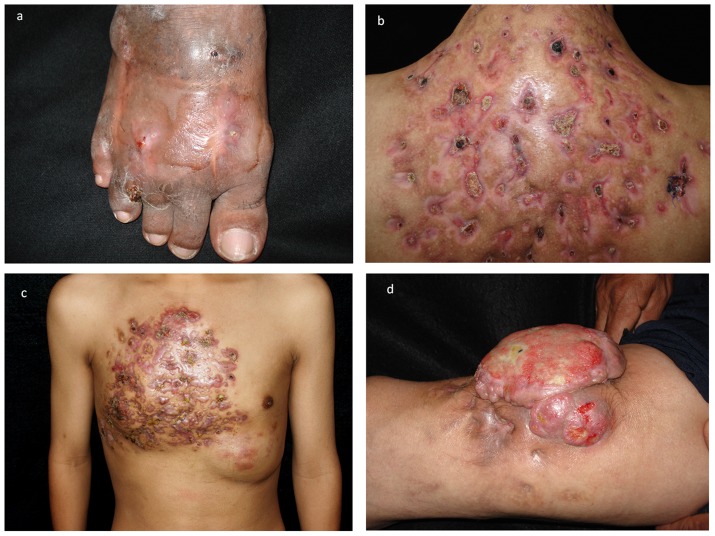
a) Foot mycetoma due to *A. madurae*. b) Back mycetoma with multiple sinuses caused by *N. brasiliensis*. c) Extensive mycetoma in an adolescent due to *N. brasiliensis*. d) Exophytic or tumoral mycetoma caused by *Fusarium solani* complex.

Lower limbs were affected in 341 cases (70.74%). Three hundred one cases (62.44%) occurred in the foot, 70 cases (14.52%) affected the upper limbs (36 cases on the hands [7.46%] and 34 on the arms [7.05%]). The trunk was involved in 49 cases (10.16%), 38 (7.88%) of those included back and shoulders. All cases involving multiple sites were associated with multiple traumatic inoculations. In regards to clinical presentation, mycetomas were classified as follows: 468 cases as tumor-like with draining sinuses (97.1%); eight cases as tumor–like without sinuses (1.65%); four cases as verrucous plaque (0.82%) and two as cystic form (0.41%). Eight patients (1.6%) presented lymphatic spread from the original mycetoma lesion: six from the foot to the inguinal area and two from the back to the axillary region. (Figure 3) One patient presented with two mycetomas, each with a distinct causative agent: the one affecting the right foot was caused by *Madurella mycetomatis*, and the second one affecting the left foot was caused by *Fusarium solani* complex.

Etiological agents were identified in 472/482 cases (98.34%). The agent was found in all of the actinomycetoma cases (n = 444 cases). In 430 cases (89.2%) the microorganisms were isolated and identified, and the remaining 14 cases were classified according to the grains observed during direct examination and/or histopathologic analysis. Two cases had double concurrently causative agents: *N. asteroides s. l.*+*N. brasiliensis* [14] and *N. brasiliensis*+*A. madurae*. Thirty-eight cases were eumycetomas and in 30/38 cases the etiological agents were isolated. Of the remaining seven cases (four hyaline and three melanized-type) only grain observation at direct microscopy was detected without identifying the causative fungi (Table 2) [15], [16].

**Table 2:** Etiologic agents of actinomycetoma and eumycetoma.

Etiologic agent	Number (%)
**Actinomycetomas**	Total: 444 (92.11)
*Nocardia brasiliensis*	377 (78.21)
*Nocardia asteroides* complex	4 (0.82)
*Nocardia otitidiscaviarum* (Syn: *Nocardia caviae*) [14] *	2 (0.41)
*Nocardia* spp	4 (0.82)
*Actinomadura madurae*	36 (7.46)
*Actinomadura pelletieri*	2 (0.41)
*Streptomyces somaliensis*	3 (0.62)
*A. madurae* grains (Identified by KOH and biopsy)	6 (1.24)
*Nocardia* spp. grains (Identified by KOH and biopsy)	8 (1.65)
Double etiology	
*N. brasiliensis+N. asteroides s.l.* [14]	1 (0.20)
*N. brasiliensis+A. madurae*	1 (0.20)
**Eumycetomas**	Total: 38 (7.88)
Black grains (total)	26 (5.39)
*Madurella mycetomatis*	15 (3.11)
*Trematospheria grisea* (Syn. *Madurella grisea*)	4 (0.82)
*Medicopsis romeroi* (Syn. *Pyrenochaeta romeroi*)	1 (0.20)
*Exophiala jeanselmei*	1 (0.20)
*Cladophialophora bantiana* (CBS 16363) [15] *	1 (0.20)
*Cladophialophora mycetomatis* (CBS 122637) [16] *	1 (0.20)
Not identified. (Confirmed by observation of black grains with KOH and biopsy)	3 (0.62)
Hyaline grains (white or yellowish): total	12 (2.48)
*Scedosporium boydii* (Syn. *Pseudallescheria boydii*)	3 (0.62)
*Fusarium solani* complex (CBS: 135554)	2 (0.41)
*Acremonium* sp	1 (0.41)
*Aspergillus nidulans*	1 (0.20)
*Microsporum canis*	1 (0.20)
Not identified	4 (0.82)

* [14], [15], [16] previously reported.

## Discussion

Mycetoma is a chronic granulomatous disease generally affecting low-income people including agricultural workers, peasants, or rural workers laboring with limited or no protective garments and soiled tools. The majority of cases (62%) described in this report affected the foot, supporting previous reports [3], [5], [6], [10] and one meta-analysis [11] that described foot as the most common site of infection (68.7% of cases). Since mycetoma presented most often on the feet of individuals living in the Indian endemic region, it explains why initial reports mentioned it as “Madura foot” [11], [17], [18], [19]. Due to the predilection for feet, mycetoma control could be achieved by using appropriate footwear and clothing that protects the limbs. However, it should be emphasized that people living in endemic regions sometimes wear open-toed shoes, mainly due to the warm climate and therefore are less protected against potential trauma [1], [5], [20], [21].

Mycetoma is associated with high morbidity and low mortality; however, the socioeconomic impact is significant; therefore patients are unable to work, resulting in decreased family income. In addition, treatments are expensive and difficult to maintain due to prolonged course of the disease. Almost all countries located in the “mycetoma belt” do not provide free quality health services or medical insurance [5], [20], [21].

The classical clinical presentation of mycetoma should lead to simple diagnosis based on the identification of a swelling zone with multiple sinus tracts; however, there is a significant lack of information between patients and clinicians, leading to delayed diagnosis and late referral to hospital, and consequently inadequate therapeutic response [5], [7], [8].

This study examined nearly 500 mycetoma cases from a single public hospital, helping to control for variables and facilitated the isolation and identification of the causative agents (the causative agent was classified in most of the cases). Ninety two percent were actinomycetoma and 8% were eumycetoma, in accordance with previous studies [10]. This result differed from one report [6] that identified 3.5% eumycetoma cases this is probably due to the difficult diagnosing these cases until they are finally referred to specialty hospitals. Reports from Latin America [22], [23], [24], [25] and, particularly, studies conducted in Mexico show a predominance of actinomycetoma, in contrast to those made in Africa, India, and Asia where cases of eumycetoma predominate [17], [20], [26], [27], [28], [29]. This epidemiological difference can be explained by differences in climate and other environmental factors. The effect of climate is observed in mycetoma cases reported in India [17], [18], [19] where the majority of actinomycetoma occur in the northern region, where the climate is subtropical and has a higher annual rainfall; while, eumycetomas occur more often in the southern where the climate is dry tropical, has a low relative humidity, and more constant temperatures. In Mexico, eumycetomas occur in drier areas. This study provides a more accurate number of cases of mycetoma in Mexico (73 new cases per-year) [6], we believe that mycetoma remains difficult for clinicians to diagnose, for that several cases may be under diagnosed and therefore underreported [1], [3], [11].

In our study, we observed that mycetoma primarily affects men, with a male∶female ratio of almost 3∶1 in our study, in concordance with a previous report about mycetoma incidence in Mexico [6]. This male predominance of micetoma can be attributed to occupational and hormonal aspect [8], [9]. The role of hormones in disease susceptibility may be explained by the few cases of mycetoma in children and the rapid growth of the lesions and increased in severity during pregnancy. Interestingly, the observed change of male∶female ratio in cases of mycetoma caused by *A. madurae* (male∶female ratio of 1∶2.2) is partly due to this microorganism is not affected by progesterone and testosterone as with *Nocardia brasiliensis* [8], [9].

Figure 1 shows mycetoma is most prevalent in the third decade of life (63.26%), which represents the most productive ages.

The mean age was 34.5 years, similar to observations were made by van de Sande [7]. Some cases were reported in elderly, however we must consider that the infection may have started many years ago, suggesting that these individuals may have acquired the disease in youth. Moreover, only 4% of the cases were reported in patients <18 years old similar to previously reported studies [6], [30]. The percentage of children infected in our study (and other reports [6], [30]) differed from a report by Fahal *et al.* [31] that described a 15% infection rate (n = 722) in children in Sudan, this was probably due to their outdoor work activities. However, the same study reported trauma in only 22.5% patients suggesting that different mechanisms of infection that deserve to be clarified [1], [31].

The main clinical presentation was tumor-like with draining sinuses; cases presented as tumor-like without sinuses and cystic form were all eumycetomas; verrucous-plaque presentation was rare, the last one is very important since its differential diagnosis include verrucous-tuberculosis, chromoblastomycosis and nontuberculous mycobacterial diseases. Although lower limbs (predominantly feet) were most commonly affected by mycetoma in our population (similar to the majority of previous reports [6], [10], [25]) it is interesting that the trunk was affected in about 10% of cases (predominantly back and shoulders). A previous Mexican report [6] described and incidence rate of the trunk in 19% of the cases, which was significantly different from the 1.4% rate described for these cases in Sudan [11]. These differences in anatomic regions affected may be explained due to occupation differences; patients in Mexico usually carry wood, sugarcane, or diverse materials on their backs. Mycetoma affecting the trunk should be considered of poor prognosis because of the proximity of lungs, spinal cord, and viscera [32]. It is also important to emphasize that cases presenting with multiple infections were described in immunocompetent patients that suffered multiple traumatic inoculations. Cases associated with lymphatic spread (1.65%) are typically seen in immunocompromised patients (malnutrition, immunosuppressants, malignant tumors, and chronic alcoholism).

*Nocardia* spp. was the main etiological agent (82.32% of cases), being *N. brasiliensis* the predominant species (78.21%). The strains were identified using phenotypic tests that lead to the identification of two more species: *N. asteroides* complex and *N. otitidiscaviarum* (formerly *N. caviae*) [14], [33]. Molecular biology techniques such as PCR and sequencing of 16S rRNA and the *hsp65* gene allow the correct identification and classification of *Nocardia* species [13]. For example, *N. mexicana*, *N. harenae*, and *N. takedensis* were isolated from Mexican patients [34], [35], [36]. The second most common causative agent was *A. madurae*; distributed worldwide [11], which is easily identified due to the larger (1–3 mm) white-yellowish, soft, wide-fringed border grains. Regardless, *A. madurae* is more difficult to isolate than *Nocardia* species, which regularly grow in rich culture media such as Lowenstein-Jensen, BHI-agar. Morphologic analyses leads to the identification of the causative agent, however, confirmation is carried out using phenotypic and temperature tests [12]. Other etiologic agents include *A. pelletieri* and *S. somaliensis* commonly found in Africa and Asia. Remarkably, the two cases presented with mixed infections following traumatic inoculation were due to *N. brasiliensis*+*N. asteroides s.l.* and *N. brasiliensis*+*A. madurae*, respectively, both presented in immunocompetent patients [14].

The causal agents of eumycetoma represented only 8% of our series, melanized fungi was the most commonly observed (26 cases, 5.39%). Of these, *Madurella mycetomatis* was the foremost isolated fungi, found in 15 cases, and also considered responsible for 25% of cases worldwide, mainly in Africa and Asia [11]. Some cases have a well-defined history of trauma (*e.g.*, thorn pricks) prior to mycetoma development; however, in some cases a well-defined traumatic event was not identified, de Hoog [37] recently noted that *M. mycetomatis* is a close relative to dung-inhabiting fungi and suggested that the natural habitat of this fungus could therefore also be dung; trauma or repeated contact with cattle dung could act as an adjuvant for inoculation of causative agents of mycetoma. Identification of *Madurella* species has been hampered by the absence of sporulation leading to confusion during the identification process. The use of molecular techniques for the identification of specific regions and genes (*e.g.*, rRNA, ITS, parcial β-tubulin gene, RNA polymerase II subunit 2 gene) has defined *Madurella* species as a cryptic complex belonging to the order Sordariales, consists primarily of *M. mycetomatis*, *M. fahalii*, *M. tropicana*, and *M. pseudomycetomatis* [38]. Identification of the infecting agent is critical since differences in thermal adaptation and susceptibility to antifungal agents exist between strains. *M. grisea* has been reclassified and now belongs to the order Pleosporales and named *Trematospheria grisea*. It should be noted that the latter was more frequently reported as a cause of mycetoma than *M. mycetomatis* in a recent report from Mexico [6]; however, difficulties in morphological and phenotypic identification could have led to confusion. Other melanized fungi further characterized by molecular biology techniques was *Exophiala jeanselmei* and *Cladophialophora bantiana*, which are common agents of phaeohyphomycosis [15]; and *Cladophialophora mycetomatis*, considered new specie [16]. Regarding hyaline fungi, *Scedosporium boydii* (Syn: *Scedosporium apiospermum*, *Pseudallescheria boydii*), was the foremost isolated strain (similar to other studies) [6], [11], [24], [26]. Mycetomas due to *Fusarium* have been previously described [39], [40], we found *F. solani* in two cases; interestingly, one of them was microscopically classified as *F. chlamydosporum*, but was reclassified using molecular biology as part of the *Fusarium solani* complex (CBS 135554). One case resulted from infection with *Aspergillus nidulans*, an agent rarely reported [41] and identified morphologically for the presence of Hülle cells. A case resulting from *Microsporum canis* infection was classified as pseudomycetoma [42] because of the rarity of the agent as a mycetoma pathogen and usually development as consequence of a chronic tinea capitis, typically seen in immunocompromised patients in the absence of traumatic inoculation [42], [43].

The study has limitations inherent to its design, however, provides important information about the status of mycetoma in Mexico. The study results can be generalized only to our population (Mexico); although the geographical areas studied has similarities with other world regions in terms of climate, distribution of etiologic agents and sociocultural conditions.

Mycetoma fulfills all the criteria of a neglected tropical disease [20], [21], [44]. It is extremely important to monitor cases and their causative agents, as a mean to understand the epidemiology of the disease, and to establish interventions for prevention, treatment and rehabilitation.

## Supporting Information

Checklist S1 STROBE Statement list in reports of cross sectional studies is completed and attached.
(DOC)
