# Supplementary material for: Mycetoma: Experience of 482 Cases in a Single Center in Mexico
Source: PLoS Negl Trop Dis. 2014 Aug 21;8(8):e3102. doi: 10.1371/journal.pntd.0003102 (PMC4140667; doi:10.1371/journal.pntd.0003102)
Supplement: Checklist S1 — STROBE Statement list in reports of cross sectional studies is completed and attached. (DOC) [file pntd.0003102.s001.doc]

STROBE Statement—Checklist of items that should be included in reports of ***cross-sectional studies***

|  | Item No | Recommendation |
| --- | --- | --- |
| **Title and abstract** | 1 | (*a*) Indicate the study’s design with a commonly used term in the title or the abstract  This is a cross-sectional study in nature, and is specified in the title and in the abstract. |
|  |  | (*b*) Provide in the abstract an informative and balanced summary of what was done and what was found.  Done, the abstract is balanced and showed the prevalence and characteristics of mycetoma in different regions of Mexico. |
| Introduction | | |
| Background/rationale | 2 | Explain the scientific background and rationale for the investigation being reported  Mexico is one of the countries that have the highest incidence of mycetoma in the world. In the introduction it is highlighted and it is determined that the information will contribute to the knowledge of the disease. |
| Objectives | 3 | State specific objectives, including any prespecified hypotheses  The main objective of the study is to provide epidemiological, clinical and microbiological data of mycetoma in Mexico. |
| Methods | | |
| Study design | 4 | Present key elements of study design early in the paper  We provide in the introduction section, some data pertaining to the study design that will guide the work flow. |
| Setting | 5 | Describe the setting, locations, and relevant dates, including periods of recruitment, exposure, follow-up, and data collection  In this retrospective analysis of the database and clinical records of the Mycology Department of the Dermatology Service at the General Hospital of Mexico, patients were enrolled between January 1980 and December 2013 (33 years). We included all mycetoma cases confirmed by microscopic observation of grains on direct examination with 10% potassium hydroxide (KOH), saline solution, and lugol solution. |
| Participants | 6 | Give the eligibility criteria, and the sources and methods of selection of participants  General epidemiologic and clinical data were extracted from complete clinical, records. |
| Variables | 7 | Clearly define all outcomes, exposures, predictors, potential confounders, and effect modifiers. Give diagnostic criteria, if applicable  The study describes accurately the clinical and microbiological characteristics of the cases presented. Diagnostic criteria for mycetoma (clinical, microbiological and histologic) are presented and completed. |
| Data sources/ measurement | 8* | For each variable of interest, give sources of data and details of methods of assessment (measurement). Describe comparability of assessment methods if there is more than one group  The culture media, the molecular and histological techniques are described entirely. There is only one group according to study design. |
| Bias | 9 | Describe any efforts to address potential sources of bias  Due to the study design, patient selection was made in a given period. The clinical records selected were those that contained all data required. Incomplete medical records were discarded. |
| Study size | 10 | Explain how the study size was arrived at  We collect all medical records that met the criteria for inclusion in a given period. |
| Quantitative variables | 11 | Explain how quantitative variables were handled in the analyses. If applicable, describe which groupings were chosen and why  We accurately describe the clinical, microbiological, histological and molecular findings of 482 medical records, no comparative analyses were done. |
| Statistical methods | 12 | Describe all statistical methods, including those used to control for confounding  Descriptive statistics were used to analyze the data. |
|  |  | (*b*) Describe any methods used to examine subgroups and interactions  Only descriptive statistics were used to analyze the data |
|  |  | (*c*) Explain how missing data were addressed  Those medical records which showed loss of data were excluded from the study. Due to the study design, data may not be found in all clinical records. |
|  |  | (*d*) If applicable, describe analytical methods taking account of sampling strategy  Not applicable |
|  |  | (*e*) Describe any sensitivity analyses  Not applicable. |
| Results | | |
| Participants | 13* | Report numbers of individuals at each stage of study—eg numbers potentially eligible, examined for eligibility, confirmed eligible, included in the study, completing follow-up, and analysed  Clinical records of 482 patients with mycetoma were included in this cross-sectional study. |
|  |  | (b) Give reasons for non-participation at each stage  Clinical records not included in the study were those who showed inconsistencies or were incomplete. |
|  |  | (c) Consider use of a flow diagram  Not applicable according to study flow |
| Descriptive data | 14* | Give characteristics of study participants (eg demographic, clinical, social) and information on exposures and potential confounders  On table 1, we describe the demographic and clinical characteristics of the patients. |
|  |  | (b) Indicate number of participants with missing data for each variable of interest  Those medical records which showed loss of data were excluded from the study. Due to the study design, data may not be found in all clinical records. |
| Outcome data | 15* | Report numbers of outcome events or summary measures  Not applicable due to the study design |
| Main results | 16 | Give unadjusted estimates and, if applicable, confounder-adjusted estimates and their precision (eg, 95% confidence interval). Make clear which confounders were adjusted for and why they were included  Not applicable |
|  |  | (*b*) Report category boundaries when continuous variables were categorized  Ranges for quantitative variables are mentioned on the text |
|  |  | (*c*) If relevant, consider translating estimates of relative risk into absolute risk for a meaningful time period  Not applicable |
| Other analyses | 17 | Report other analyses done—eg analyses of subgroups and interactions, and sensitivity analyses  Not applicable |
| Discussion | | |
| Key results | 18 | Summarise key results with reference to study objectives  In the discussion section, we make reference to the findings of the study and correspond to the objectives set out in the study. |
| Limitations | 19 | Discuss limitations of the study, taking into account sources of potential bias or imprecision. Discuss both direction and magnitude of any potential bias  The study has limitations inherent in its design, however, provides important information about the status of mycetoma in Mexico. |
| Interpretation | 20 | Give a cautious overall interpretation of results considering objectives, limitations, multiplicity of analyses, results from similar studies, and other relevant evidence  The study findings are in accordance with those reported in the international literature, also, the importance of epidemiological surveillance and further study to establish a global casuistry are highlighted |
| Generalisability | 21 | Discuss the generalisability (external validity) of the study results  The study results can be generalized to our population (Mexico), although the geographical areas studied has similarities with other world regions in terms of climate and distribution of etiologic agents. |
| Other information | | |
| Funding | 22 | Give the source of funding and the role of the funders for the present study and, if applicable, for the original study on which the present article is based  The present study had no funding sources of any kind or funders. |

*Give information separately for exposed and unexposed groups.

**Note:** An Explanation and Elaboration article discusses each checklist item and gives methodological background and published examples of transparent reporting. The STROBE checklist is best used in conjunction with this article (freely available on the Web sites of PLoS Medicine at http://www.plosmedicine.org/, Annals of Internal Medicine at http://www.annals.org/, and Epidemiology at http://www.epidem.com/). Information on the STROBE Initiative is available at www.strobe-statement.org.
